# The genome sequence of the Grey Dagger, *Acronicta psi* (Linnaeus, 1758)

**DOI:** 10.12688/wellcomeopenres.18711.1

**Published:** 2023-01-27

**Authors:** Gavin R. Broad, Natural History Museum Genome Acquisition Lab, Darwin Tree of Life Barcoding collective, Wellcome Sanger Institute Tree of Life programme, Wellcome Sanger Institute Scientific Operations: DNA Pipelines collective, Tree of Life Core Informatics collective, Darwin Tree of Life Consortium

**Affiliations:** 1 Department of Life Sciences, Natural History Museum, London, UK

**Keywords:** Acronicta psi, Grey Dagger, genome sequence, chromosomal, Lepidoptera

## Abstract

We present a genome assembly from an individual male *Acronicta psi* (the Grey Dagger; Arthropoda; Insecta; Lepidoptera; Noctuidae). The genome sequence is 405 megabases in span. The whole assembly is scaffolded into 31 chromosomal pseudomolecules, including the assembled Z sex chromosome. The mitochondrial genome has also been assembled and is 15.4 kilobases long.

## Species taxonomy

Eukaryota; Metazoa; Ecdysozoa; Arthropoda; Hexapoda; Insecta; Pterygota; Neoptera; Endopterygota; Lepidoptera; Glossata; Ditrysia; Noctuoidea; Noctuidae; Acronictinae; *Acronicta*; *Acronicta psi* (Linnaeus, 1758) (NCBI:txid987865).

## Background

*Acronicta psi,* Grey Dagger, is a common and widespread moth in Britain and Ireland, found throughout Europe, North Africa, the Near East and Central Asia. The larvae are polyphagous, feeding on a variety of trees and shrubs, but particularly Rosaceae, in woodland, gardens and a variety of other habitats ( Waring *et al.,* 2017). The caterpillars feed in the summer and autumn with adults on the wing, at least in Britain, usually from late April through July, although sometimes with a second generation in the autumn.

The Latin and English names both refer to the prominent black streak on the tornal area of the forewing, resembling the Greek ψ (psi), or a dagger shape. Although a distinctive feature at the genus level, *A. psi* adults are confusingly similar to Dark Dagger, *Acronicta tridens* (Denis and Schiffermüller). They are only really separable by genitalia examination (nicely illustrated by Townsend *et al.,* 2011). Males of the two species are easily separable by the number of projections on the ventral surface of the valva. The genitalia of the specimen sequenced were checked and retained.

Both species are widespread and polyphagous although, unlike the adults, the larvae of Dark and Grey Dagger are very different in appearance. *A. psi* has been used as an *exemplar generalis* moth species in investigating the different responses of specialists and generalists to elevated levels of tannin compounds found in leaves ( Roslin & Salminen, 2008). Unlike some Oak- ( *Quercus*) feeding specialists, the tannin Vescalagin has a severe impact on the growth of *A. psi* larvae. This genome adds to a rapidly increasing number of genomes for Lepidoptera and should be very useful to researchers interested in the hugely significant radiation of the family Noctuidae.

## Genome sequence report

**Figure 1.**
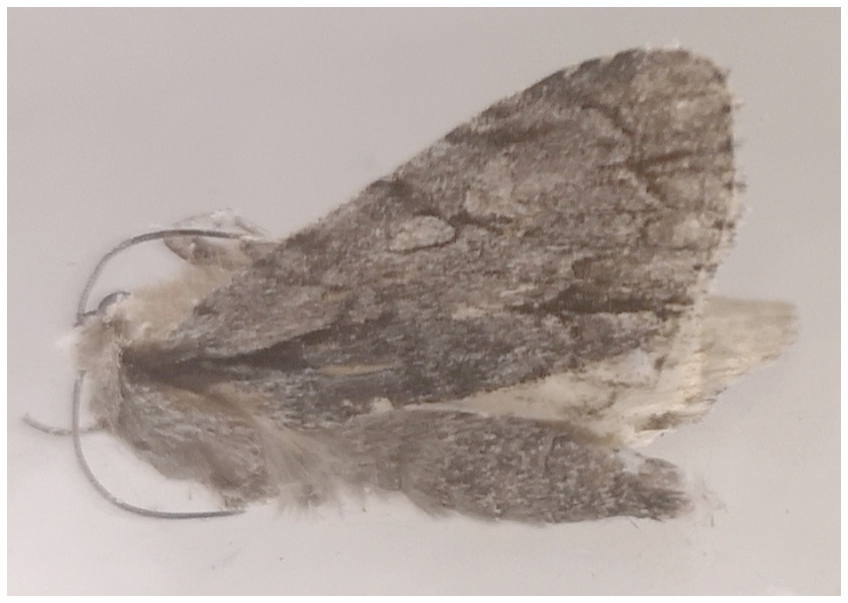
Photograph of the *Acronicta psi* (ilAcrPsix1) specimen used for genome sequencing taken during preservation and processing.

**Figure 2.**
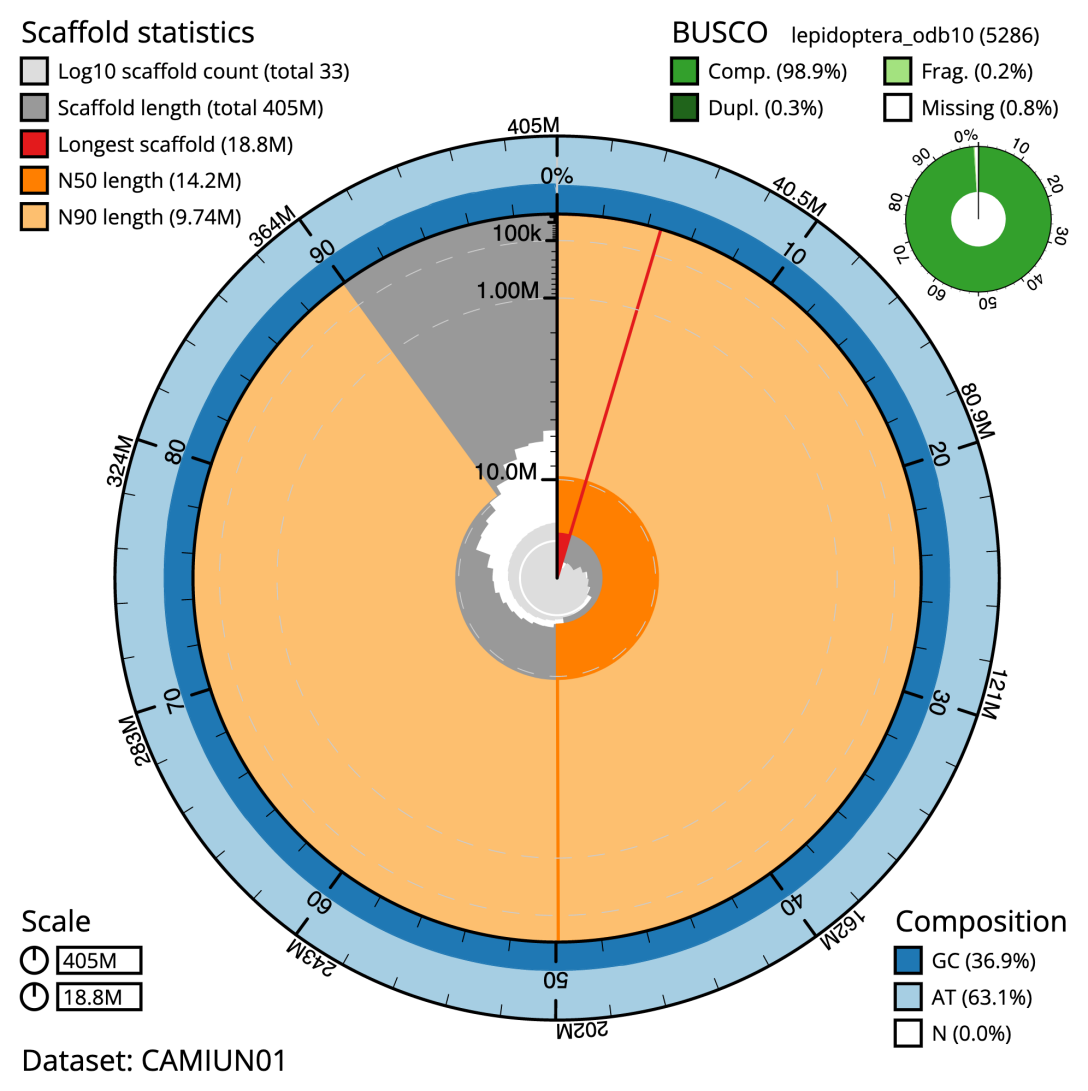
Genome assembly of *Acronicta psi*, ilAcrPsix1.1: metrics. The BlobToolKit Snailplot shows N50 metrics and BUSCO gene completeness. The main plot is divided into 1,000 size-ordered bins around the circumference with each bin representing 0.1% of the 404,700,304 bp assembly. The distribution of scaffold lengths is shown in dark grey with the plot radius scaled to the longest scaffold present in the assembly (18,777,983 bp, shown in red). Orange and pale-orange arcs show the N50 and N90 chromosome lengths (14,236,034 and 9,736,517 bp), respectively. The pale grey spiral shows the cumulative scaffold count on a log scale with white scale lines showing successive orders of magnitude. The blue and pale-blue area around the outside of the plot shows the distribution of GC, AT and N percentages in the same bins as the inner plot. A summary of complete, fragmented, duplicated and missing BUSCO genes in the lepidoptera_odb10 set is shown in the top right. An interactive version of this figure is available at https://blobtoolkit.genomehubs.org/view/ilAcrPsix1.1/dataset/CAMIUN01/snail.

The genome was sequenced from one male *Acronicta psi* ( Figure 1) collected from a garden in Tonbridge, UK (51.19, 0.29). A total of 45-fold coverage in Pacific Biosciences single-molecule HiFi long reads was generated. Primary assembly contigs were scaffolded with chromosome conformation Hi-C data. Manual assembly curation corrected one missing join, reducing the scaffold number by 3.03%. The final assembly has a total length of 404.7 Mb in 32 sequence scaffolds with a scaffold N50 of 14.2 Mb ( Table 1). The whole assembly sequence was assigned to 31 chromosomal-level scaffolds, representing 30 autosomes, and the Z sex chromosome ( Figure 2– Figure 5; Table 2). Chromosome-scale scaffolds confirmed by the Hi-C data are named in order of size. The genome assembly for *Acronicta aceris* (GCA_910591435) ( Boyes *et al.,* 2021) was used to identify the sex chromosome. While not fully phased, the assembly deposited is of one haplotype. Contigs corresponding to the second haplotype have also been deposited. The assembly has a BUSCO v5.3.2 ( Manni *et al.*, 2021) completeness of 98.6% (single 98.6%, duplicated 0.3%) using the lepidoptera_odb10 reference set.

**Table 1.** Genome data for *Acronicta psi*, ilAcrPsix1.1.

Project accession data	
Assembly identifier	ilAcrPsix1.1
Species	*Acronicta psi*
Specimen	ilAcrPsix1
NCBI taxonomy ID	987865
BioProject	PRJEB55133
BioSample ID	SAMEA11025022
Isolate information	male thorax (PacBio sequencing), head (Hi-C)
Assembly metrics *	
Consensus quality (QV)	67.7 (Benchmark: ≥50)
*k*-mer completeness	100 (Benchmark: ≥95%)
BUSCO **	C:98.9%[S:98.6%,D:0.3%], F:0.2%,M:0.8%,n:5,286 (Benchmark: C ≥ 95%)
Percentage of assembly mapped to chromosomes	100% (Benchmark: ≥95%)
Sex chromosomes	Z (Benchmark: localised homologous pairs)
Organelles	Mitochondrial genome assembled (Benchmark: complete single alleles)
Raw data accessions	
PacificBiosciences SEQUEL II	ERR10033482
Hi-C Illumina	ERR10038430
Genome assembly	
Assembly accession	GCA_946251955.1
*Accession of alternate haplotype*	GCA_946251945.1
Span (Mb)	404.7
Number of contigs	33
Contig N50 length (Mb)	14.2
Number of scaffolds	32
Scaffold N50 length (Mb)	14.2
Longest scaffold (Mb)	18.8

* Assembly metric benchmarks are adapted from column VGP-2020 of “Table 1: Proposed standards and metrics for defining genome assembly quality” from ( Rhie *et al.*, 2021). ** BUSCO scores based on the lepidoptera_odb10 BUSCO set using v5.3.2. C = complete [S = single copy, D = duplicated], F = fragmented, M = missing, n = number of orthologues in comparison. A full set of BUSCO scores is available at https://blobtoolkit.genomehubs.org/view/ilAcrPsix1.1/dataset/CAMIUN01/busco.

**Figure 3.**
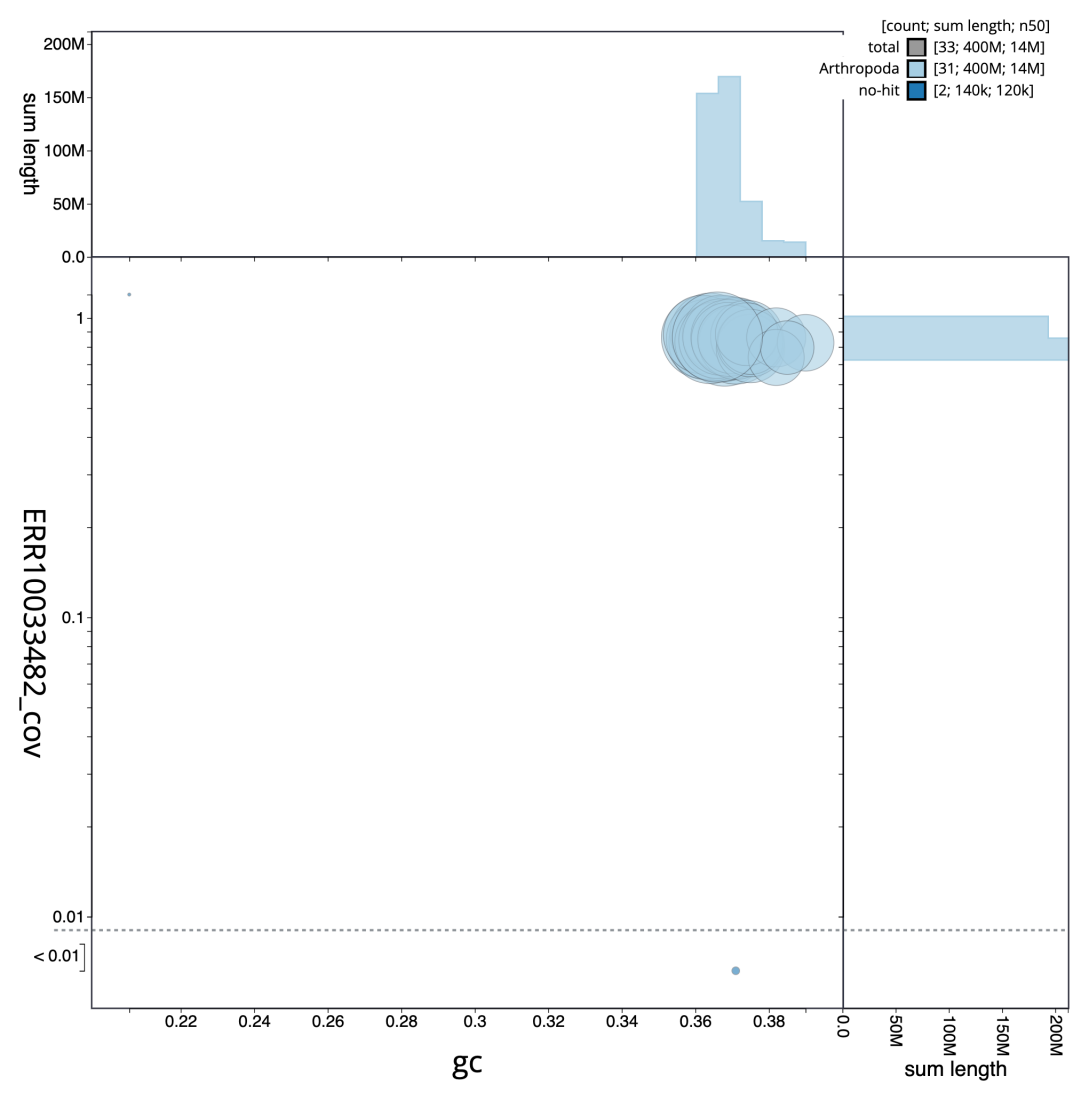
Genome assembly of *Acronicta psi*, ilAcrPsix1.1: GC coverage. BlobToolKit GC-coverage plot. Chromosomes are coloured by phylum. Circles are sized in proportion to scaffold length. Histograms show the distribution of scaffold length sum along each axis. An interactive version of this figure is available at https://blobtoolkit.genomehubs.org/view/ilAcrPsix1.1/dataset/CAMIUN01/blob.

**Figure 4.**
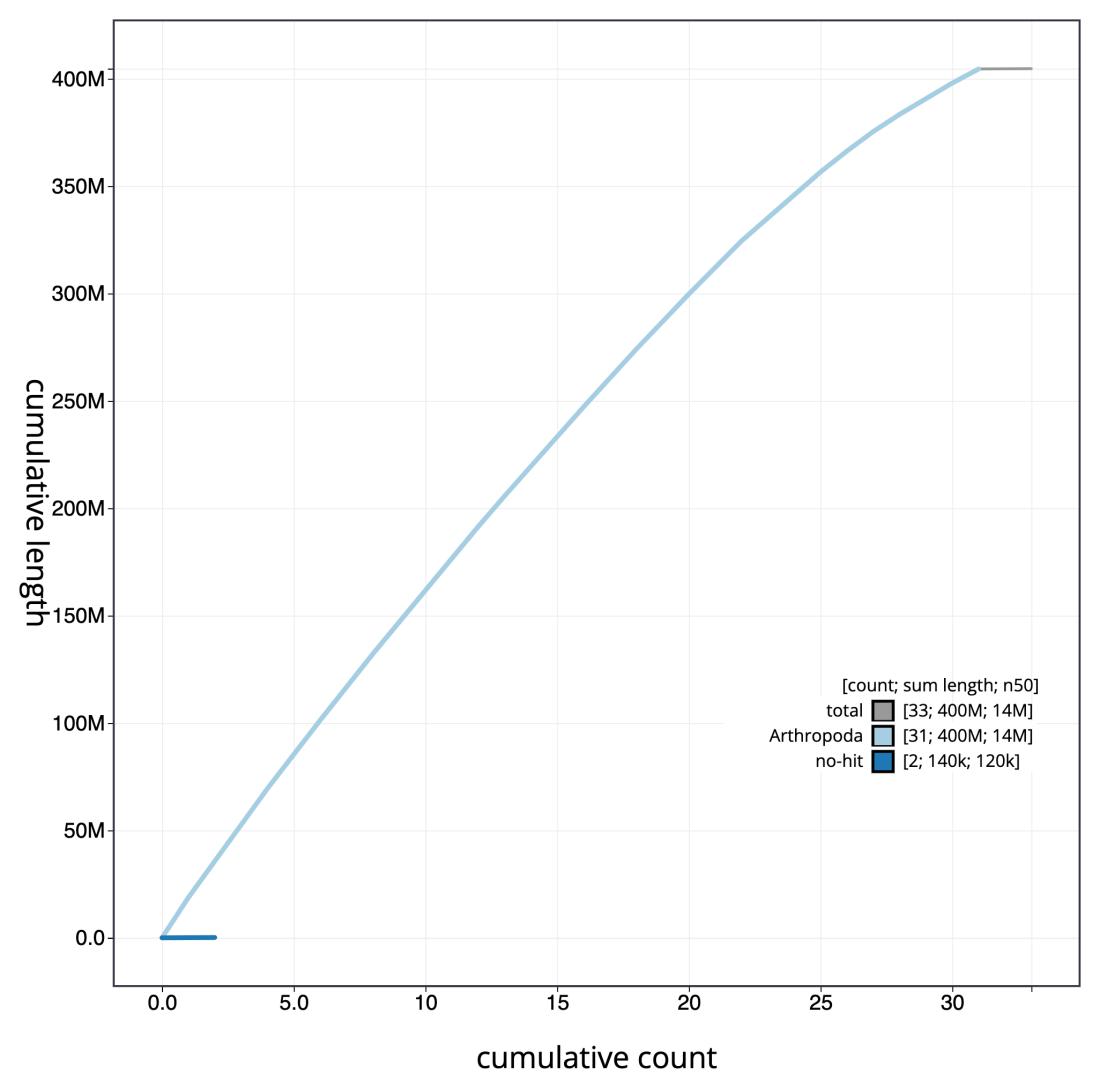
Genome assembly of *Acronicta psi*, ilAcrPsix1.1: cumulative sequence. BlobToolKit cumulative sequence plot. The grey line shows cumulative length for all chromosomes. Coloured lines show cumulative lengths of scaffolds assigned to each phylum using the buscogenes taxrule. An interactive version of this figure is available at https://blobtoolkit.genomehubs.org/view/ilAcrPsix1.1/dataset/CAMIUN01/cumulative.

**Figure 5.**
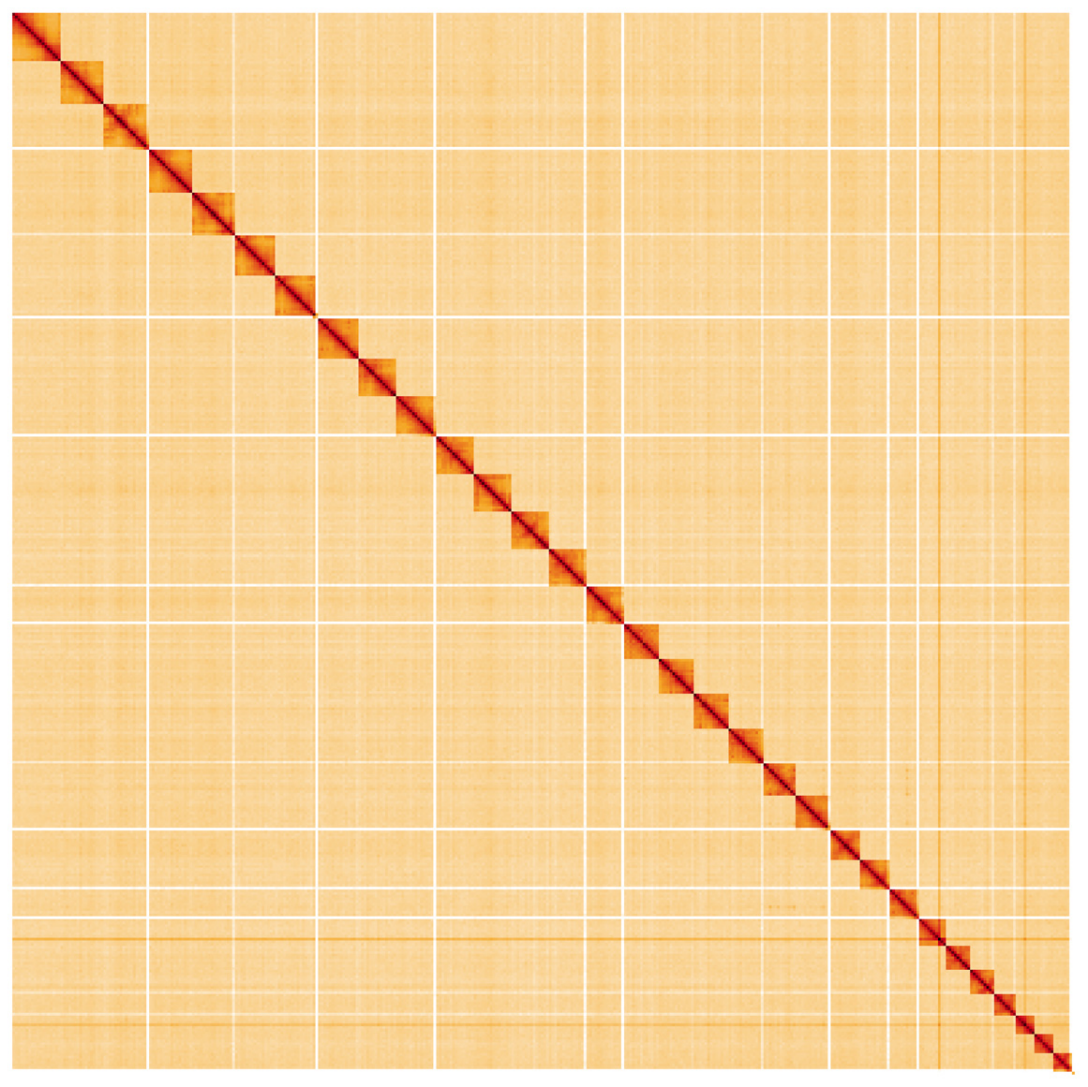
Genome assembly of *Acronicta psi*, ilAcrPsix1.1: Hi-C contact map. Hi-C contact map of the ilAcrPsix1.1 assembly, visualised using HiGlass. Chromosomes are shown in order of size from left to right and top to bottom. An interactive version of this figure may be viewed at https://genome-note-higlass.tol.sanger.ac.uk/l/?d=ZNuV1Z0qSX2nydjV_mmC4Q.

**Table 2.** Chromosomal pseudomolecules in the genome assembly of *Acronicta psi*, ilAcrPsix1.

INSDC accession	Chromosome	Size (Mb)	GC%
OX276469.1	1	16.87	36.8
OX276470.1	2	16.97	37.1
OX276471.1	3	16.86	36.6
OX276472.1	4	16.1	36.9
OX276473.1	5	15.68	36.2
OX276474.1	6	15.57	36.4
OX276475.1	7	15.31	36.6
OX276476.1	8	14.83	36.4
OX276477.1	9	14.83	36.8
OX276478.1	10	14.82	36.3
OX276479.1	11	14.8	36.8
OX276480.1	12	14.24	36.2
OX276481.1	13	13.93	36.5
OX276482.1	14	13.9	36.7
OX276483.1	15	13.7	36.6
OX276484.1	16	13.48	36.7
OX276485.1	17	13.33	37.1
OX276486.1	18	13.05	36.9
OX276487.1	19	12.72	37.1
OX276488.1	20	12.62	36.9
OX276489.1	21	12.12	37.4
OX276490.1	22	10.88	37
OX276491.1	23	10.77	37.5
OX276492.1	24	10.46	37.5
OX276493.1	25	9.74	37.5
OX276494.1	26	9.07	37.4
OX276495.1	27	8.01	38.2
OX276496.1	28	7.35	39
OX276497.1	29	7.21	38.2
OX276498.1	30	6.57	38.5
OX276468.1	Z	18.78	36.6
OX276499.1	MT	0.02	20.8
-	-	0.12	37.1

## Methods

### Sample acquisition and nucleic acid extraction

A male *Acronicta psi* specimen (ilAcrPsix1) was collected from a garden in Tonbridge, Kent, UK (latitude 51.19, longitude 0.29), using a light trap and then snap-frozen at -80°C. The specimen was collected and identified by Gavin Broad (Natural History Museum).

DNA was extracted from thorax tissue of ilAcrPsix1 at the Wellcome Sanger Institute (WSI) Scientific Operations core from the whole organism using the Qiagen MagAttract HMW DNA kit, according to the manufacturer’s instructions.

### Sequencing

Pacific Biosciences HiFi circular consensus DNA sequencing libraries were constructed according to the manufacturers’ instructions. DNA sequencing was performed by the Scientific Operations core at the WSI on Pacific Biosciences SEQUEL II (HiFi) instrument. Hi-C data were also generated from head tissue of ilAcrPsix1 using the Arima v2 kit and sequenced on the Illumina NovaSeq 6000 instrument.

### Genome assembly

Assembly was carried out with Hifiasm ( Cheng *et al.*, 2021) and haplotypic duplication was identified and removed with purge_dups ( Guan *et al.*, 2020). The assembly was scaffolded with Hi-C data ( Rao *et al.*, 2014) using YaHS ( Zhou *et al.,* 2022). The assembly was checked for contamination as described previously ( Howe *et al.*, 2021). Manual curation was performed using HiGlass ( Kerpedjiev *et al.*, 2018) and Pretext ( Harry, 2022). The mitochondrial genome was assembled using MitoHiFi ( Uliano-Silva *et al.*, 2021), which performed annotation using MitoFinder ( Allio *et al.*, 2020). The genome was analysed and BUSCO scores were generated within the BlobToolKit environment ( Challis *et al.*, 2020). Table 3 contains a list of all software tool versions used, where appropriate.

**Table 3.** Software tools and versions used.

Software tool	Version	Source
BlobToolKit	3.4.0	Challis *et al.*, 2020
Hifiasm	version 0.16.1-r375	Cheng *et al.*, 2021
HIGlass	1.11.6	Kerpedjiev *et al.*, 2018
PretextView	0.2	Harry, 2022
purge_dups	1.2.3	Guan *et al.*, 2020
MitoHiFi	2.x	Uliano-Silva *et al.*, 2021
YaHS	yahs-1.1.91eebc2	Zhou *et al*., 2022

### Ethics/compliance issues

The materials that have contributed to this genome note have been supplied by a Darwin Tree of Life Partner. The submission of materials by a Darwin Tree of Life Partner is subject to the Darwin Tree of Life Project Sampling Code of Practice. By agreeing with and signing up to the Sampling Code of Practice, the Darwin Tree of Life Partner agrees they will meet the legal and ethical requirements and standards set out within this document in respect of all samples acquired for, and supplied to, the Darwin Tree of Life Project. Each transfer of samples is further undertaken according to a Research Collaboration Agreement or Material Transfer Agreement entered into by the Darwin Tree of Life Partner, Genome Research Limited (operating as the Wellcome Sanger Institute), and in some circumstances other Darwin Tree of Life collaborators.

## Data Availability

European Nucleotide Archive: *Acronicta psi*. Accession number PRJEB55133; https://identifiers.org/ena.embl/PRJEB55133 ( Wellcome Sanger Institute, 2022) The genome sequence is released openly for reuse. The *Acronicta psi* genome sequencing initiative is part of the Darwin Tree of Life (DToL) project. All raw sequence data and the assembly have been deposited in INSDC databases. The genome will be annotated using available RNA-Seq data and presented through the Ensembl pipeline at the European Bioinformatics Institute. Raw data and assembly accession identifiers are reported in Table 1. Members of the Natural History Museum Genome Acquisition Lab are listed here: https://doi.org/10.5281/zenodo.4790042. Members of the Darwin Tree of Life Barcoding collective are listed here: https://doi.org/10.5281/zenodo.4893703. Members of the Wellcome Sanger Institute Tree of Life programme are listed here: https://doi.org/10.5281/zenodo.4783585. Members of Wellcome Sanger Institute Scientific Operations: DNA Pipelines collective are listed here: https://doi.org/10.5281/zenodo.4790455. Members of the Tree of Life Core Informatics collective are listed here: https://doi.org/10.5281/zenodo.5013541. Members of the Darwin Tree of Life Consortium are listed here: https://doi.org/10.5281/zenodo.4783558.
