# Supplementary material for: The genome sequence of the Grey Dagger, Acronicta psi (Linnaeus, 1758)
Source: Wellcome Open Res. 2023 Jan 27;8:39. [Version 1] doi: 10.12688/wellcomeopenres.18711.1 (PMC10285329; doi:10.12688/wellcomeopenres.18711.1)
Supplement: Peer review history

# Peer review: Reviewer response for version 1

**DOI:** 10.21956/wellcomeopenres.20749.r57409

**Published:** 2023-06-21

**Authors:** Anyi Mazo-Vargas

**Affiliations:** 1 The George Washington University, Washington, District of Columbia, USA

The note describes the assembly of the Grey Dagger, *Acronicta psi* genome. The note is straightforward and very clear. I only have some notes about the methods:

1. The links in the ENA show transcriptomic data. Did you produce the data? If yes, from what tissue/stage? Even if the annotation is a work in process, you are publishing all the datasets together, so the authors should include some information here. 
2. Could you add more details about the parameters or a link to the scripts? There is not enough information for replication. 

Best!

Are sufficient details of methods and materials provided to allow replication by others?

Partly

Is the rationale for creating the dataset(s) clearly described?

Yes

Are the datasets clearly presented in a useable and accessible format?

Yes

Are the protocols appropriate and is the work technically sound?

Yes

Reviewer Expertise:

Evolutionary and developmental biology

I confirm that I have read this submission and believe that I have an appropriate level of expertise to confirm that it is of an acceptable scientific standard.

# Peer review: Reviewer response for version 1

**DOI:** 10.21956/wellcomeopenres.20749.r57416

**Published:** 2023-05-31

**Authors:** Sivasankaran Kuppusamy

**Affiliations:** 1 Entomology Research Institute, Loyola Collège, Chennai, India

I would like to appreciate the authors for compiling the Grey Dagger, *Acronicta psi* complete genome sequence. The authors have employed the proper software, genome assembly techniques, and whole genome sequencing methods.

 In the abstract…The first sentence can be modified as “The whole genome sequence of Grey Dagger, *Acronicta psi* (Lepidoptera: Noctuidae) was sequenced and assembled”.

**Materials and Methods**

 Page number 5, second paragraph subheading **Sequencing **last sentence can be changed as “ *Hi-C *data were also generated from head tissue of ilAcrPsix1 using Arima v2 kit and sequenced on the Ilumina NovaSeq 6000 platform ( *manufacturer company name of instrument should be given within the parenthesis*)”

 Page no. 3 Genome sequence report

 The sentence can be modified as “The whole genome was sequenced from a male specimen of *Aconicta psi……………..*

 Total sequence lengths and longest scaffold lengths were properly mentioned in the legend.

 The manuscript is well written overall, and it can be accepted for indexing.

Are sufficient details of methods and materials provided to allow replication by others?

Yes

Is the rationale for creating the dataset(s) clearly described?

Yes

Are the datasets clearly presented in a useable and accessible format?

Yes

Are the protocols appropriate and is the work technically sound?

Yes

Reviewer Expertise:

Analyzing the phylogenetic relationships of superfamily Noctuoidea moths using mitochondrial genome sequence

I confirm that I have read this submission and believe that I have an appropriate level of expertise to confirm that it is of an acceptable scientific standard.
